# An Engineered Reporter Phage for the Fluorometric Detection of *Escherichia coli* in Ground Beef

**DOI:** 10.3390/microorganisms9020436

**Published:** 2021-02-19

**Authors:** Anqi Chen, Danhui Wang, Sam R. Nugen, Juhong Chen

**Affiliations:** 1 Department of Food Science, Cornell University, Ithaca, NY 14853, USA; ac939@cornell.edu (A.C.); dw554@cornell.edu (D.W.); jhchen@vt.edu (J.C.); 2 Department of Food Science and Technology, The Ohio State University, Columbus, OH 43210, USA; 3 Department of Biological Systems Engineering, Virginia Tech, Blacksburg, VA 24061, USA

**Keywords:** bacteria detection, engineered bacteriophages, fluorescent assay, beta-galactosidase, ground beef

## Abstract

Despite enhanced sanitation implementations, foodborne bacterial pathogens still remain a major threat to public health and generate high costs for the food industry. Reporter bacteriophage (phage) systems have been regarded as a powerful technology for diagnostic assays for their extraordinary specificity to target cells and cost-effectiveness. Our study introduced an enzyme-based fluorescent assay for detecting the presence of *E. coli* using the T7 phage engineered with the *lacZ* operon which encodes beta-galactosidase (β-gal). Both endogenous and overexpressed β-gal expression was monitored using a fluorescent-based method with 4-methylumbelliferyl β-d-galactopyranoside (MUG) as the substrate. The infection of *E. coli* with engineered phages resulted in a detection limit of 10 CFU/mL in ground beef juice after 7 h of incubation. In this study, we demonstrated that the overexpression of β-gal coupled with a fluorogenic substrate can provide a straightforward and sensitive approach to detect the potential biological contamination in food samples. The results also suggested that this system can be applied to detect *E. coli* strains isolated from environmental samples, indicating a broader range of bacterial detection.

## 1. Introduction

Foodborne pathogens are important food safety issues in both developed and developing countries [1]. In the United States, close to 10 million food-related illnesses have been reported each year, resulting in excessive burdens on public health and major impediments to socio-economic growth [2,3]. Since bacterial pathogen infection has been the key cause of foodborne diseases, the identification of these microbes in food samples is of importance in ensuring food safety. Among all pathogens, *Escherichia coli* (*E. coli*) has been considered one of the most common causes of hundreds of reported foodborne outbreaks in the United States [4]. According to the Centers for Disease Control and Prevention (CDC), *E. coli* contamination has been found in a wide range of food and environmental samples including raw and pasteurized fluid milk, cheese, and drinking water [5]. Thus, developing improved methods that are practical for the food industry for implementation is necessary.

In order to address the increasing demand for rapid and accurate bacterial detection, a number of advanced tools have been established, including simple polymerase chain reaction (PCR), oligonucleotide DNA microarray, enzyme-linked immunosorbent assay (ELISA) and surface plasmon resonance (SPR) [6,7,8]. Despite their reliability and precision, these approaches either require significant equipment and sample preparation or are comparatively expensive for total material and labor expense [8,9,10]. As an alternative, bacteriophages (phages) present ideal tools for bacterial detection. Phages are viruses that recognize their host cells with extraordinary specificity which allows them to be incorporated as a tool for detecting the presence of target bacterial cells. In addition, phages are able to self-replicate and therefore generate progeny phages within a short amount of time, allowing an acceleration of signal amplification which can help achieve a lower detection limit without any pre-enrichment steps [11]. Another important benefit of using phages for bacterial detection is their ability to differentiate between live and dead cells [12]. Given that phage infection and reproduction only occur in viable bacterial hosts, phage-based detection approaches ensure that only viable cells are detected [13].

In recent years, phages have been incorporated into many of the rapid methods used for the detection of bacteria in food matrices [14,15]. To develop bacterial biosensors with unique properties, genetically engineered phages have been innovated in multiple studies [2,16,17]. Among the most commonly used methods is the “reporter phage” detection system, in which recombinant genes are introduced into the wild-type phage genome to allow expression only when phage–host infection occurs. Once expressed, the reporter gene provides an indicative signal for the detection of bacteria using colorimetric, fluorescent, or bioluminescent signals that can be produced only after viable cells are infected [12,18,19,20,21,22]. β-gal, a well-known bacterial enzyme encoded by the *lacZ* operon, has been frequently used as a biomarker to assess the presence of a variety of organisms, such as *E. coli*, for its widespread presence in both eukaryotes and prokaryotes.

**Figure 1:**
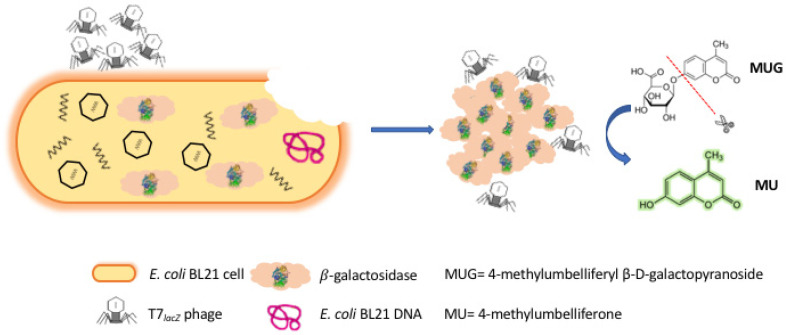
Schematic representation of the T7 engineered phage (T7*_lacZ_* phage)-based fluorescent assay of *E. coli* in ground beef. T7 phages were engineered to overexpress the reporter enzyme β-gal. Phage infection induces the production and release of intracellular and phage-induced β-gal. The substrate 4-methylumbelliferyl β-D-galactopyranoside (MUG) was metabolized by β-gal to generate the fluorescent product 4-methylumbelliferone (MU).

The assay introduced in this study aims to rapidly detect generic *E. coli* as a contamination indicator in ground beef, which was used here as the representative complex food matrix for it being a common contamination associated with common foodborne outbreaks. Briefly, the principle of this strategy was based on the fluorometric measurement of both endogenous and overexpressed β-gal enzyme released by engineered T7 phages (T7*_lacZ_* phages) infecting its target bacteria *E. coli* (Figure 1). The use of T7*_lacZ_* phages functioned as an important tool to identify living *E. coli* cells and allowed the overexpression of the reporter enzyme β-gal in aqueous solution to increase the sensitivity of the assay. It also reduced the risk of false-positive outcomes associated with nonviable bacteria since phages can only replicate in specific viable hosts. Additionally, the use of T7*_lacZ_* phages coupled with a fluorogenic method allowed for an easy, sensitive, and cost-effective detection of *E. coli*.

## 2. Materials and Methods

### 2.1. Chemicals, Materials, and Instruments

The fluorogenic substrate 4-methylumbelliferyl β-D-galactopyranoside (4-MUG) was obtained from Sigma-Aldrich (St. Louis, MO, USA). Milli-Q water (EMD Millipore, Billerica, MA, USA) at 25 °C with 18 MΩ/cm resistivity used throughout all the experiments. A BioTek spectrophotometer (Winooski, VT, USA) with a Gen5^TM^ Microplate Reader was used to determine the *E. coli* concentrations at OD_600_ (optical density measured at a wavelength of 600 nm) and the fluorophore synthesis level was monitored at an emission/excitation wavelength of 365/460 nm since the cleavage of 4-MUG by the β-gal enzyme yields the fluorescent molecule 4-MU that emits light at 460 nm when excited by 365 nm light [23]. The 96-well clear microplates and 96-well black microplates were purchased from Corning (Corning, NY, USA) for measuring bacterial concentrations and the fluorescence intensities, respectively.

### 2.2. Bacterial Strains and Culture Media

The model bacteria for this research *E. coli* BL21 (ATCC 25922) was inoculated into 20 mL of Luria–Bertani broth (LB broth, 10 g tryptone, 5 g yeast extract, and 10 g NaCl in 1 L of distilled water, pH 7.2) and incubated at 37 °C overnight with 200 rpm agitation. Then, the *E. coli* culture was centrifuged at 7000× *g* for 2 min, washed twice with phosphate-buffered saline (PBS buffer: 100 mM Na_2_HPO_4_, 18 mM KH_2_PO_4_, 27 mM KCl and 1.37 M NaCl, pH 7.4) and resuspended in PBS buffer. *E. coli* BL21 concentration was measured by colony counting on LB agar plates (10 g tryptone, 5 g yeast extract, 10 g NaCl, and 15 g agar in 1 L of distilled water, pH 7.2).

### 2.3. T7 Engineered Phage

T7 engineered phage construction for the overexpression of β-gal was first introduced in our previous study [24]. Briefly, the *lacZ* construct, which is 3075 base pairs in size, was constructed in a pUC57 plasmid (GenScript, Piscataway, NJ, USA) with the *lacZ* operon. The amplification of *lacZ* was performed using Phusion PCR kit (Ipswich, MA, USA) and PCR products were purified using PCR Purification Kit from Qiagen (Hilden, Germany). EcoRI and HindIII were the two restriction enzymes used for digestion. Then, by using T4 DNA ligase (Promega, Madison, WI, USA), the construct was successfully inserted into the genome vector T7Select415 from EMD Millipore (Billerica, MA, USA). Then, the T7*_lacZ_* phages were obtained by assembling the engineered *lacZ*-carrying T7*_lacZ_* genome. To confirm β-gal overexpression by T7*_lacZ_* phage, a control phage (T7_control_) without the ability to overexpress intracellular β-gal during phage infection was generated by replacing the inserted *lacZ* construct with an *S Tag.* Both T7*_lacZ_* and T7_control_ were propagated in *E. coli* BL21 and plated. The plaques were isolated and verified for the correct size insert using a Phusion PCR kit with the T7Select Up and Down primers. A double agar overlay plaque assay was used to quantify the plaque-forming unit (PFU/mL) of the engineered phages). In short, 100 µL of phages culture was first added into a 3 mL melted top LB agar layer containing 200 µL of overnight *E. coli* culture. The top agar was then poured onto a solid LB agar layer, which was incubated for 3 h at 37 °C before plaque counting.

### 2.4. Detection of E. coli BL21 in Buffer Solution by T7_lacZ_ Phages

Overnight *E. coli* culture was serially diluted (10-fold) in sterile LB and each time was washed once with sterile PBS buffer. The final reaction mixture contained 100 µL of *E. coli* (0, 10^1^, 10^2^, 10^3^, 10^4^, and 10^5^ CFU/mL), 100 µL of T7*_lacZ_* phages (1 × 10^4^ PFU/mL), and 200 µL of 2.5 mM MUG in 600 µL LB. The solutions were incubated at 37 °C with agitation at 200 rpm. The fluorescence intensity of each sample (200 µL) was measured at 1 h intervals over a period of 8 h using a plate reader. Fluorophore (4-methylumbelliferone (MU)) synthesis was monitored at emission/excitation wavelengths of 365/460 nm, respectively. LB broth containing T7*_lacZ_* phage and MUG only was included as a negative control.

### 2.5. Preparation of Ground Beef Samples

The organic ground beef sample used in this study contained 85% lean and 15% fat (net wt. 453 g) and was purchased from a local retailer (Ithaca, NY, USA). The manufacturer claimed that no hormones or antibiotics was added to the ground beef, which was inspected by the USDA (United States Department of Agriculture). One portion (25 g) of raw ground beef was blended with 225 mL of PBS buffer in a sterile Stomacher strainer bag and agitated for 1 min in a Lab blender Stomacher 400 circulator (Seward, Norfolk, UK). The mixture was then filtered with a 0.22 µm pore-size syringe filter (Corning Life Science, Corning, NY, USA) to reduce potential interference from other bacteria in the ground beef, as tested previously in other food matrices [2]. The homogenized ground beef juice was centrifuged in a 50 mL centrifuge tube at 7000× *g* for 5 min. After centrifugation, the supernatant was carefully aspirated and transferred into clean 50 mL sterile polypropylene conical tubes (Corning Inc., Corning, NY, USA).

### 2.6. Detection of E. coli BL21 in Ground Beef Using T7_lacZ_ Phages

An overnight culture of *E. coli* BL21 was inoculated in beef mixture at final concentrations of 10^1^, 10^2^, 10^3^, 10^4^, and 10^5^ CFU/mL. The final reaction matrix contained 100 µL of *E. coli*, 100 µL of T7*_lacZ_* phage (1 × 10^4^ PFU/mL), and 200 µL MUG (2.5 mM) in 600 µL beef juice. Beef juice containing phage lysate and MUG substrate only was used as a negative control. Then, all the samples were incubated at 37 °C with agitation at 200 rpm for 5, 6, 7, and 8 h. Then, the fluorescence intensity of each sample (200 µL) was measured using a plate reader as described previously.

### 2.7. Detection of Other E. coli Strains in Ground Beef Using T7_lacZ_ Phages

Ten isolated *E. coli* strains other than *E. coli* BL21 were selected randomly from four different beef farms. All strains, including *E. coli* BL21, were grown in 20 mL of LB broth overnight at 37 °C. Then, the overnight *E. coli* culture was centrifuged at 7000× *g* for 2 min, washed twice with PBS buffer, and resuspended in PBS. The *E. coli* concentrations were measured by plate counting before all strains were diluted to 10^6^ CFU/mL. The final reaction mixture contained 100 µL of *E. coli*, 100 µL of T7*_lacZ_* phages (1 × 10^4^ PFU/mL), and 200 µL of MUG (2.5 mM) in 600 µL LB. After incubation at 37 °C for 3.5 h, the fluorescence intensity of each sample (200 µL) was measured using a plate reader as described previously.

### 2.8. Statistical Analysis

All experiments were conducted in triplicate and independently for each condition. All data were evaluated for statistical significance using a *t*-test and are presented as the mean ± standard deviation. The asterisks (*) indicate that the tested set of data showed a difference (*p* < 0.05) when compared to the control set.

## 3. Results

### 3.1. Principle of Detecting E. coli Using T7_lacZ_ Phages

In this study, we presented a rapid and facile fluorescence-based method for the detection of *E. coli* cells using engineered phages. As illustrated in Figure 1, once T7*_lacZ_* phages attached to the surfaces of *E. coli* cells, they injected their genome into the host bacterial cells, generated early proteins to degrade the host DNA, and allowed T7*_lacZ_* phages to take control of the cellular machinery by initiating phage biosynthesis [25,26]. While the T7*_lacZ_* phages replicated inside the host cells, the *lacZ* gene was expressed from the phage genome, and therefore, β-gal enzymes were abundantly produced. At the end of phage infection cycle, the host cells were disrupted by phage enzymes and eventually lysed, releasing reproduced T7*_lacZ_* progeny phages as well as the phage-induced reporter enzyme β-gal into the aqueous environment [27]. The evaluation of β-gal production was based on the conversion of the substrate MUG, a non-fluorescent galactosidase analogue, into the highly fluorescent molecule MU. By taking advantage of current genetic engineering techniques that have allowed the overexpression of reporter proteins driven by stronger promoters, we managed to amplify the fluorescent signals by measuring both endogenous and phage-induced β-gal activity.

### 3.2. Optimization of Phage Infection and the Enzymatic Reaction

**Figure 2:**
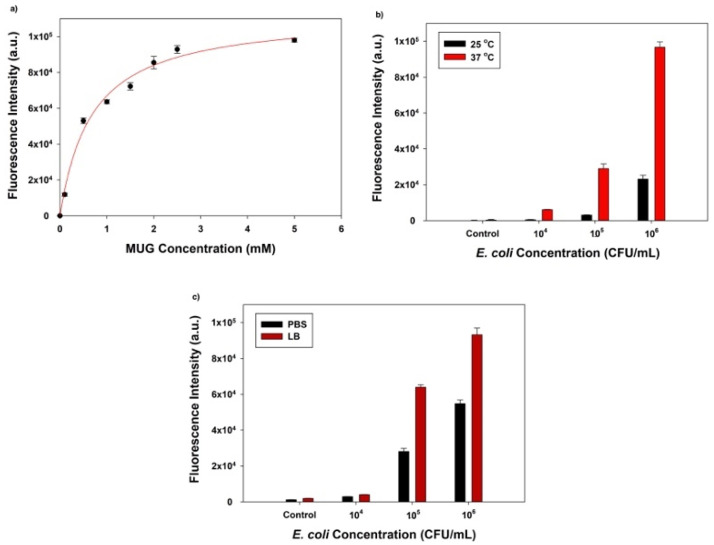
(**a**) The determination of optimal MUG concentration based on fluorescent intensities; (**b**) comparison of the fluorescence intensity obtained at two incubation temperatures (37 °C, red bars; 25 °C, black bars); (**c**) comparison of the fluorescence intensity obtained using two reaction media: phosphate-buffered saline (PBS) (black bars) and Luria–Bertani (LB) broth (red bars). The control group indicates the absence of bacteria in the reaction mixture. The values reflect the average of at least three independent biological replicates and standard deviation is shown as the error bars in each graph.

The first factor we optimized was the substrate (MUG) concentration, which was closely associated with the degree of β-gal enzymatic activity and the final fluorescence signal intensity. Before the fluorescent signal reaches saturation, an increased substrate concentration would result in a higher signal intensity if the amount of enzyme was fixed. A 25 µL aliquot of 10^4^ PFU/mL T7*_lacZ_* phages and 50 µL MUG with different concentrations (0, 0.1, 0.5, 1, 1.5, 2, 2.5, and 5 mM) in LB were used to infect 10^6^ CFU/mL of *E. coli*. After 3.5 h of incubation at 37 °C, the correlation between the fluorescence intensity and MUG concentrations suggested that the amount of fluorophore gradually increased with increasing substrate concentration but levelled off at approximately 2.5 mM (Figure 2a). Thus, MUG substrate at a concentration of 2.5 mM was selected for all the following experiments.

Another optimized factor to maximize the detection limit was the incubation temperature, 37 vs. 25 °C (room temperature). While the ideal temperature for phage reproduction and *E. coli* growth is 37 °C, setting up reactions at room temperature is more accessible in settings with limited resources. To examine whether the incubation temperature is significantly important, we compared the fluorescent intensities detected at different *E. coli* cell concentrations (10^4^, 10^5^, and 10^6^ CFU/mL). For the negative control group, we incubated T7*_lacZ_* phages and MUG only in LB liquid (no *E. coli*). Aliquots containing 25 µL T7*_lacZ_* phages (10^4^ PFU/mL) and 50 µL of 2.5 mM MUG were used to infect *E. coli* at 10^4^, 10^5^, and 10^6^ CFU/mL for 3.5 h at 25 and 37 °C, respectively. As expected, at both temperatures (25 and 37 °C), higher bacterial concentrations resulted in higher fluorescence signals (Figure 2b). More importantly, due to *E. coli* proliferation, the fluorescence intensities of all treatment groups were significantly higher after the samples being incubated at 37 °C than at 25 °C. Thus, for subsequent experiments, 37 °C was designated as the optimized incubation temperature.

The reaction medium (PBS vs. LB broth) was another factor we considered to optimize the assay sensitivity. Similarly, aliquots containing 25 µL T7*_lacZ_* phages (10^4^ PFU/mL) and 50 µL of 2.5 mM MUG were used to infect *E. coli* cells at various concentrations (10^4^, 10^5^, and 10^6^ CFU/mL). As expected, the fluorescence intensity was positively correlated with the bacterial concentration in both PBS and LB (Figure 2c). However, the samples incubated in LB consistently generated a higher signal than PBS regardless of the *E. coli* concentration. Thus, for optimization purposes, LB was chosen over PBS for the following experiments.

### 3.3. Detection of E. coli BL21 Using Control Phages (T7_control_ Phages), Engineered Phages (T7_lacZ_ Phages), and No Phages

**Figure 3:**
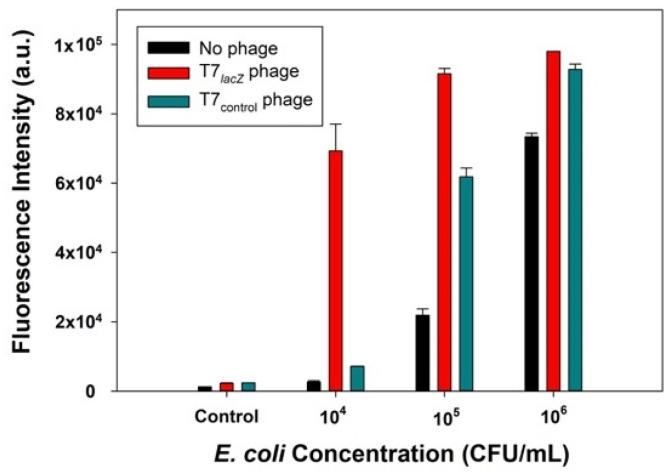
Comparison of the fluorescence intensity measured using T7*_lacZ_* phages (red bars), T7_control_ phages (green bars), and no phages (dark blue bars). Control group indicates the absence of bacteria in the reaction mixture. The values reflect the average of at least three independent biological replicates and standard deviation is shown as the error bars.

We hypothesized that the signal generated from bacterial detection with the T7*_lacZ_* phage would be significantly higher than that obtained from the T7_control_ phage and no phage groups. To test this hypothesis, T7*_lacZ_* phages (10^4^ PFU/mL), T7_control_ phages (10^4^ PFU/mL), and no phages were used to infect *E. coli* at various concentrations (10^4^, 10^5^, and 10^6^ CFU/mL) for 3.5 h at 37 °C. Phages incubated with the substrate MUG in the absence of bacterial cells was the negative control. The results indicated that when the incubation conditions were identical, the T7*_lacZ_* phage group showed a significantly higher fluorescence signal than the T7_control_ phage and no phage group at each tested *E. coli* concentration (Figure 3). This implied that the amount of β-gal produced using T7*_lacZ_* phages was substantially enhanced compared to that generated in the other two groups. Interestingly, the signal from the T7_control_ phages was also remarkably higher than that in the no phage group at higher *E. coli* concentrations (10^5^ and 10^6^ CFU/mL). In addition, the sample containing 10^6^ CFU/mL of *E. coli* in the group without phages also showed a high signal (Figure 3).

### 3.4. Detection of E. coli BL21 in Buffer Solution by T7_lacZ_ Phages

**Figure 4:**
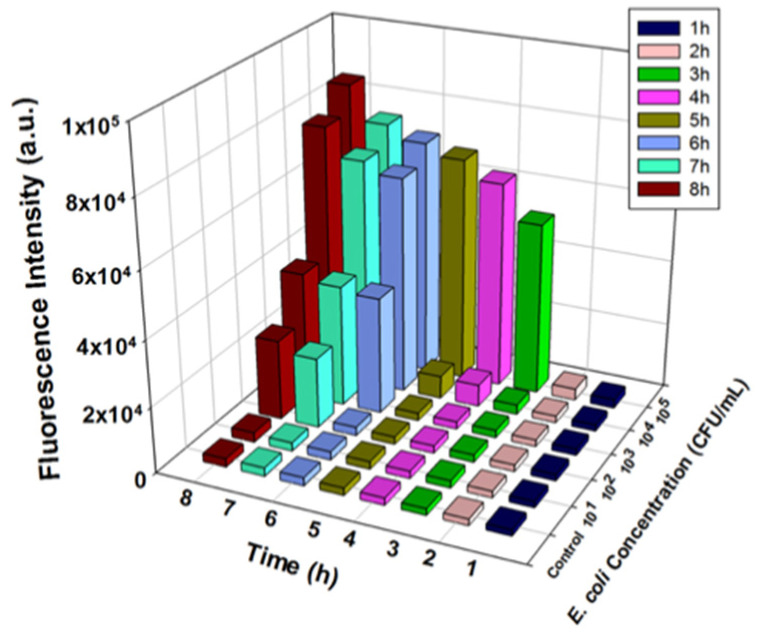
Fluorescence intensity measured from T7*_lacZ_* phages infecting *E. coli* in LB at varying concentrations (0, 10, 10^2^, 10^3^, 10^4^, and 10^5^ CFU/mL) after incubating for 8 h at 1 h intervals at 37 °C. The control group indicates the absence of bacteria in the reaction mixture. Data represent the mean of a minimum of three independent replicates.

Once we optimized the reaction factors and demonstrated the effectiveness of using the T7*_lacZ_* phages, *E. coli* detection at different concentrations (0, 10^1,^ 10^2^, 10^3^, 10^4^, and 10^5^ CFU/mL) was performed. The amount of fluorescent product generated by β-gal-dependent MUG hydrolysis was quantified at 1 h intervals over a period of 8 h. The detected fluorescent signals increased with increasing *E. coli* concentrations and the incubation duration (Figure 4 and Figure S1a–h). Expectedly, the control group, which lacked *E. coli*, generated a consistently low signal throughout the 8 h incubation. Although the fluorescence level of the incubation matrix containing 10 CFU/mL of *E. coli* was consistently indistinguishable from that of the control group, signals from the samples with 10^2^, 10^3^, 10^4^, and 10^5^ CFU/mL of *E. coli* were all significantly higher than that from the control at different timepoints during the 8 h incubation. For example, after 2 h of incubation, only samples with 10^5^ CFU/mL of *E. coli* were noticeably different (Figure S1b), while after 7 h, samples with 10^2^–10^5^ CFU/mL of bacteria were all able to be detected (Figure S1g). A longer incubation time enhanced the signal at each concentration of *E. coli* due to an increase in enzymatic reaction between the fluorescent substrate and reporter enzyme β-gal (Figure 4). Therefore, based on the above observations, we concluded that the detection of 100 CFU/mL *E. coli* in LB broth after 7 h of incubation was the maximum detection capacity of our proposed assay.

### 3.5. Detection of E. coli BL21 in Ground Beef Using T7_lacZ_ Phages

**Figure 5:**
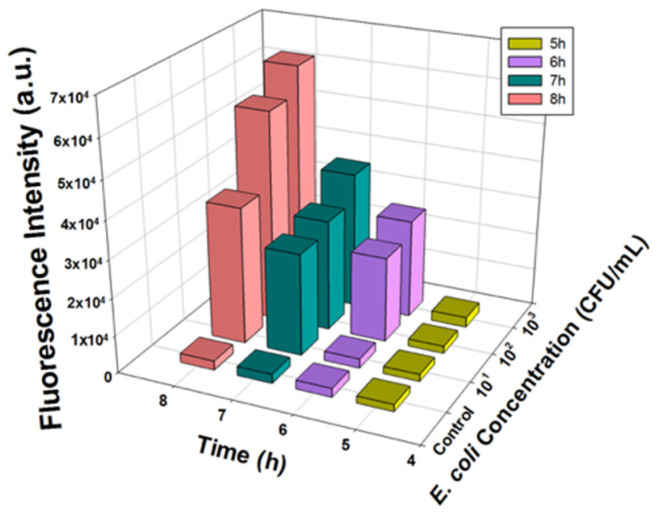
Fluorescence intensity measured from T7*_lacZ_* phages infecting *E. coli* in beef juice at varying concentrations (0, 10, 10^2^, 10^3^, 10^4^, and 10^5^ CFU/mL) after incubating for 5, 6, 7 and 8 h at 37 °C. Data represent the means of a minimum of three independent replicates.

Ground beef was first homogenized in PBS, and then the filtered sterile beef juice was intentionally inoculated with *E. coli* BL21. The fluorescence intensities of the samples with different *E. coli* concentrations (10, 10^2^, and 10^3^ CFU/mL) and the negative control group (no *E. coli*) were quantified after 5, 6, 7, and 8 h of incubation. A similar response in fluorescence production was observed in the ground beef samples, as shown in Figure 5. As predicted, the control group maintained a consistently low background intensity, indicating the barely detectable signal throughout the reaction. Additionally, as the incubation time increased, lower *E. coli* concentrations could be detected: 5 and 6 h of incubation allowed the detection of *E. coli* at concentrations of 10^3^ CFU/mL and 10^2^ CFU/mL, respectively (Figure S2a,b). The lowest concentration detectable by this assay in ground beef samples was that of an initial inoculum to be as low as 10 CFU/mL after approximately 7 h (Figure S2c).

### 3.6. Detection of Other E. coli Strains in Ground Beef Using T7_lacZ_ Phages

**Figure 6:**
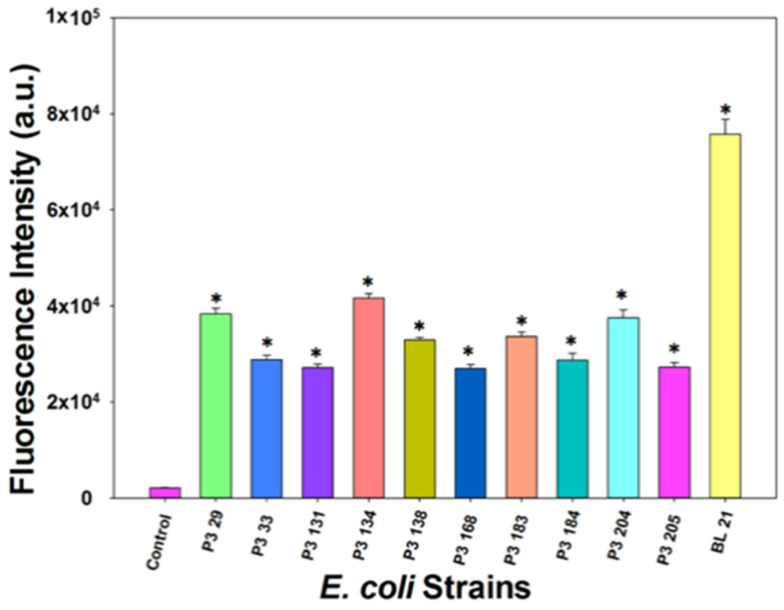
Fluorescence intensity measured from T7*_lacZ_* phages infecting different *E. coli* strains in ground beef juice after 3.5 h of incubation at 37 °C. Control group indicates the absence of bacteria in the reaction mixture. The values reflect the average of at least three independent biological replicates and standard deviation is shown as the error bars. Asterisks (*) represent groups that are significantly different (*p* < 0.05) from the control.

*E. coli* outbreaks associated with ground beef products reflect the link to cattle as a major source of human infections [28]. To demonstrate the broad application range of our developed assay, we randomly selected and isolated ten *E. coli* strains from four local cattle farms. Overnight cultures of these selected strains were diluted to 10^6^ CFU/mL in LB and treated with T7*_lacZ_* phages (10^4^ PFU/mL). The fluorescence intensities after the samples were incubated at 37 °C for 3.5 h are shown in Figure 6. The negative control contained no bacterial cells, while the positive control contained *E. coli* BL21. The fluorescence intensity of each strain was significantly higher than that of the negative control, indicating that our proposed assay is applicable to the detection of other *E. coli* strains isolated from environmental samples.

## 4. Discussion

There is a growing demand for the rapid detection of bacterial contamination in foods to ensure the food safety and public health. Bacteriophages have been extensively used as the valuable method for the detection of bacteria for their high specificity, fast infection cycle, and inexpensive preparation [29]. The T7 bacteriophage and its host, *E. coli*, are thoroughly investigated models used in scientific studies. The fact that T7 bacteriophage genome is relatively simple has allowed multiple genetic modifications by inserting different reporter genes. A number of phage-based bacteria detection approaches have been discussed and improved for higher bacteria detection sensitivity [14,17,29,30]. To extend the applicability of this system, our study offers a straightforward and rapid strategy using engineered T7 phages to detect *E. coli* in ground beef. The value of this assay is that it could be used to broadly detect *E. coli* as an indicator of potential food contamination and therefore developed into a food safety application.

### 4.1. Optimization of Phage Infection and the Enzymatic Reaction

A number of factors, including substrate (MUG) concentration, incubation temperature, and reaction media, can influence the effectiveness and efficiency of our proposed method. It is not to a great surprise that LB media provided a higher sensitivity than the PBS buffer (Figure 2c). As a complex medium, LB broth contains both yeast extract and tryptone, highly nutritious components that contributed to bacterial proliferation and therefore optimized the environment for phage infection [31].

One potential factor that might also affect the sensitivity and/or change the detection limit of our assay is T7*_lacZ_* phage concentration. Our previous work studying the matrix of phage amplification indicated a dynamic interaction between the proliferation of bacterial cells and phage reproduction: the overall amount of enzymes released largely relies on the number of bacterial cells infected as well as the phage concentration [17,24]. Specifically, increased phage infection results in the enhanced transcription of *lacZ*, resulting in an increased production of β-gal. However, a high initial phage concentration often induces the rapid lysis of *E. coli* cells, thereby inhibiting the reproduction of uninfected bacterial cells. Therefore, instead of optimizing the T7*_lacZ_* phage concentration, we decided to use the concentration determined earlier (10^4^ PFU/mL) throughout this study.

### 4.2. Detection of E. coli BL21 Using Control Phages (T7_control_ Phages), Engineered Phages (T7_lacZ_ Phages), and No Phages

The T7*_lacZ_* phages were designed for two purposes in our proposed system. First, as a lytic phage, T7 phage lysed the host cell and therefore released intracellular proteins (e.g., β-gal) at the end of infection cycles. Second, the engineered T7*_lacZ_* phages were designed with a *lacZ* operon encoding β-gal, which can be overexpressed and amplified the fluorescence detection signal. In order to show the feasibility of the use of the T7*_lacZ_* phage, we compared the signal obtained from T7*_lacZ_* phages, T7_control_ phages (no operon expressing β-gal), and the no phages infection of *E. coli*.

The observation that T7*_lacZ_* phages generated the highest detection signal (Figure 3) confirmed that the use of T7*_lacZ_* phages allowed excess β-gal synthesis in addition to the endogenous β-gal produced by *E. coli*. Both overexpressed and endogenous β-gal were then discharged into the reaction media and therefore amplified the final signal intensity. One interesting result was that at high *E. coli* concentrations (10^5^ and 10^6^ CFU/mL), the signal intensities of the T7_control_ group was noticeably higher than that of no phage group. This can be explained by the fact that a high number of *E. coli* cells contain more endogenous β-gal enzymes. Although *E. coli* cells lysed by T7_control_ phages are sufficient to generate a high fluorescence signal, the benefit of using T7_control_ phages was lost in the presence of T7*_lacZ_* phages when detecting bacteria at a lower concentration (10^4^ CFU/mL). Another interesting observation was the high intensity from the no phage group containing 10^6^ CFU/mL of *E. coli*. This can be explained by the proliferation of *E. coli* cells without phage infection during incubation. Since *E. coli* cells were capable of accumulating intracellular β-gal if they were not infected by phages during the incubation, it is possible that free β-gal enzymes were released from the natural bacterial cell lyses. Though limited, the diffusion of MUG through the cell membrane might also explain the high fluorescent signal detected in this particular sample.

### 4.3. Host Specificity of T7_lacZ_ Phage

One requirement for the detection of *E. coli* in complex food matrices using our proposed method is to ensure the specificity of the genetically modified T7 phages. It is known that phages specifically target a subset of bacterial strains and phage specificity heavily depends on the structure of receptors on the bacterial cell surface. More specifically, T7 phages directly attach their tail fibers to the *E. coli* cell membrane, specifically lipopolysaccharides (LPS). The specificity of our proposed assay was evaluated in our previous study by testing whether T7*_lacZ_* can infect other bacteria, including *Staphylococcus aureus* (*S. aureus*), *Salmonella enterica* (*S. enterica*), *Pseudomonas aeruginosa* (*P. aeruginosa*), and a cocktail containing all three bacterial strains plus *E. coli* BL21 [24]. The results showed that a large signal occurred only in samples containing *E. coli*. In other words, T7*_lacZ_* phages specifically target *E. coli* cells.

### 4.4. Detection of E. coli BL21 in Buffer Solution by T7_lacZ_ Phages

To test the feasibility of our proposed assay, the detection of *E. coli* was evaluated on the basis of using MUG as the substrate in this enzymatic reaction occurred only when intact *E. coli* cells were infected by T7*_lacZ_* phages. Adding T7*_lacZ_* phages and the substrate MUG at the same time enabled simultaneous enzymatic reactions and phage reproduction. Since it can detect *E. coli* at a concentration as low as 100 CFU/mL of *E. coli* after 7 h of incubation, we infer that any concentrations above 100 CFU/mL should be applicable, although concentrations higher than 10^5^ CFU/mL were not tested. For further optimization, we will optimize the desired incubation duration by examining the signals generated between 6 and 7 h at 20 min intervals to determine the minimum required incubation time.

### 4.5. Detection of E. coli BL21 and Other E. coli Strains in Ground Beef Using T7_lacZ_ Phages

Since ground beef has been implicated in a large number of foodborne outbreaks caused by pathogenic *E. coli*, the standards and regulations for monitoring its contamination with *E. coli* are very stringent. The U.S. Food Safety Inspection Service has announced a zero-tolerance threshold for the pathogenic *E. coli* O157:H7 contamination of raw meat products [28]. Thus, in this study, ground beef was used as an example to examine the effectiveness of the T7*_lacZ_* phages in detecting *E. coli* in complex food matrices. Intriguingly, this concentration was lower than the minimum detectable concentration in buffer solution after 7 h of incubation (100 CFU/mL) (Figure 4). One explanation is that beef juice has more abundant nutrients and therefore provided a more desirable matrix for bacterial growth, resulting in the high proliferation of the bacterial population. Therefore, it is reasonable to infer that the decrease in the detection limit was due to the release of an endogenous β-gal from the accumulated *E. coli*. Another important value of this assay was demonstrated by examining whether other *E. coli* strains, different from BL21, can also be infected by T7*_lacZ_* phages and generate enhanced fluorescent signal. Figure 6 suggests the feasibility of using this strategy to broadly detect *E. coli* as an indicator of potential food contamination.

Our approach can detect 10 CFU/mL of *E. coli* in 7 h with no required pre-enrichment steps. In order to achieve a lower detection limit, the following preparations can be performed in later studies to further improve the sensitivity of our method: (1) pre-enrichment, which might help reach an even lower limit of detection without extending the required incubation time; (2) the phage genome can be engineered with a stronger promoter to enhance the production of reporter enzymes that can be detected using more sensitive quantification methods.

The proposed method has been examined as effective in testing a variety of liquid samples including river water [17], drinking water, skim milk and orange juice [15,24]. The successful use of our strategy in detecting *E. coli* in ground beef, a solid food sample, indicates that it has a great potential to be applied for bacterial detection in other food matrices. One study using reporter phage ΦV10nLuc detecting the luminescent signal generated from pathogenic *E. coli* O157:H7 required a minimum of 7 h incubation to detect 5 CFU in ground beef [32], which is slightly higher than the detection limit proposed in this study. In another study, a minimum of 8 h incubation is reported for engineered T4*_lacZ_* phages to detect 10 CFU/mL *E. coli* [33]. However, a pre-enrichment step (4 h incubation in nutrient medium) is required before the phage-mediated cell lysis. Other phage-based methods require high phage concentrations for detection, such as 10^8^ PFU/mL [20,22], while the phage concentration used in this assay was 10^4^ PFU/mL, suggesting the potential to provide a cost-effective strategy that can be conveniently applied to the food industry.

## 5. Conclusions

This study offers a straightforward and rapid strategy using engineered phages to detect *E. coli* in ground beef. The value of this assay is that it could be used to broadly detect *E. coli* as an indicator of potential food contamination and therefore developed into a food safety application. Our assay also revealed that wild-type phages that can be genetically manipulated to carry a reporter gene (*lacZ*) can be expressed only when phage–host infection occurs, thus resulting in a higher signal than that generated from the assays using unmodified phages. Signal amplification by overexpressing the reporter protein β-gal by the engineered T7*_lacZ_* phages, which also infect and lyse *E. coli* cells, promotes the sensitivity of this process. This method can detect 100 CFU/mL *E. coli* in the buffer solution and 10 CFU/mL in ground beef juice samples after 7 h of incubation. In conclusion, our work suggests that the use of a fluorogenic substrate to detect β-gal activity caused by engineered phage infection is a promising path to fast and precise bacterial detection. As a proof-of-concept method, it has the potential to be used in large industrial food processing facilities.

## Data Availability

Not applicable.

## Supplementary Materials

The following are available online at https://www.mdpi.com/2076-2607/9/2/436/s1, Figure S1: Fluorescence intensity measured from T7*_lacZ_* phages infecting *E. coli* with different concentrations (0, 101, 102, 103, 104, and 105 CFU/mL) in buffer solution after (a) 1 h; (b) 2 h; (c) 3 h; (d) 4 h; (e) 5 h; (f) 6 h; (g) 7 h; and (h) 8 h of incubation at 37 °C, respectively; Figure S2: Fluorescence intensity measured from T7*_lacZ_* phages infecting *E. coli* with different concentrations (0, 10, 10^2^, 10^3^ CFU/mL) in ground beef after (a) 5 h; (b) 6 h; (c) 7 h; and (d) 8 h of incubation at 37 °C, respectively.
