# Supplementary material for: An Engineered Reporter Phage for the Fluorometric Detection of Escherichia coli in Ground Beef
Source: Microorganisms. 2021 Feb 19;9(2):436. doi: 10.3390/microorganisms9020436 (PMC7922204; doi:10.3390/microorganisms9020436)
Supplement: Supplementary file 1 [file microorganisms-09-00436-s001.pdf]

Supporting Information

An engineered reporter phage for the fluorometric detection of *Escherichia coli* in ground beef

Anqi Chen,[a] Danhui Wang,[a, b] Sam R. Nugen*[a] and Juhong Chen[a,c]

[a]Department of Food Science, Cornell University, Ithaca, NY 14853, United States

[b]Department of Food Science and Technology, The Ohio State University, Columbus, OH 43210, United States

[c]Department of Biological Systems Engineering, Virginia Tech, Blacksburg, Virginia 24061, United States

Corresponding author:

* (SRN). Email: snugen@cornell.edu

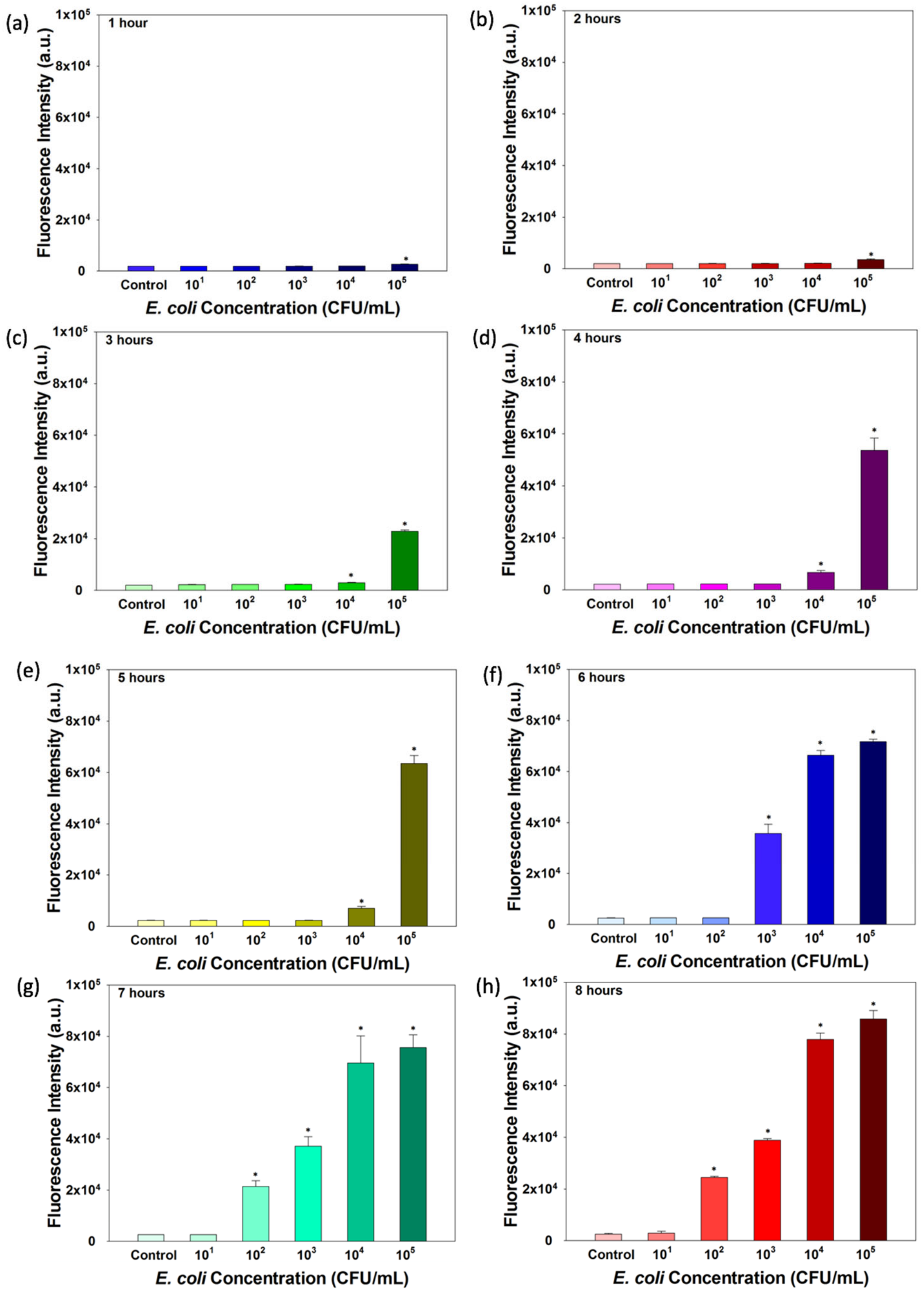

Figure S1 Fluorescence intensity measured from T7$_{lacZ}$ phages infecting *E. coli* with different concentrations (0, 10^1, 10^2, 10^3, 10^4, and 10^5 CFU/mL) in buffer solution after (a) 1 hour (b) 2 hours (c) 3 hours (d) 4 hours (e) 5 hours (f) 6 hours (g) 7 hours (h) 8 hours of incubation at 37°C, respectively.

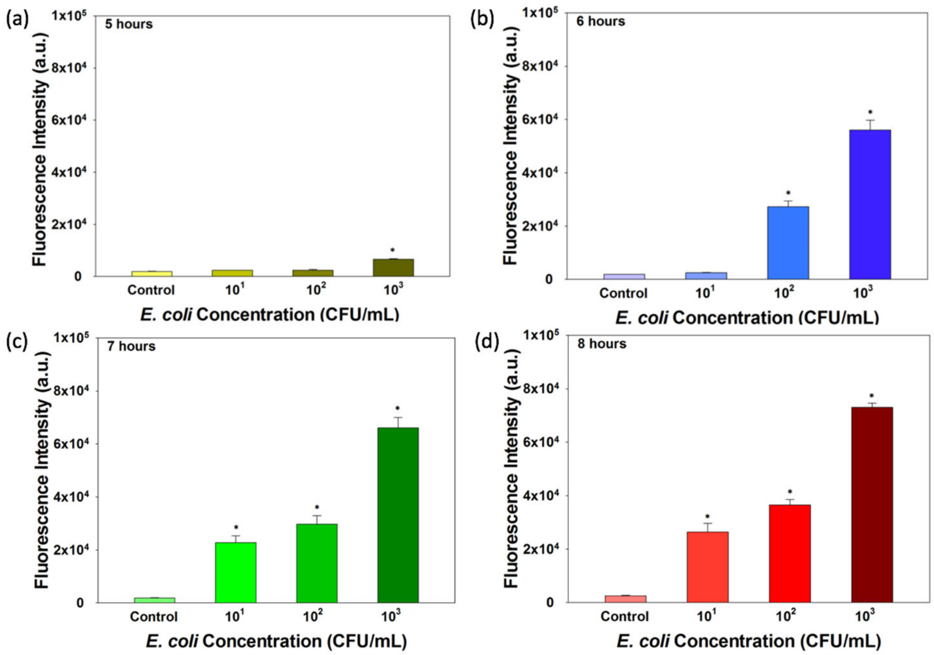

Figure S2 Fluorescence intensity measured from T7$_{lacZ}$ phages infecting *E. coli* with different concentrations (0, 10, 10^2, 10^3 CFU/mL) in ground beef after (a) 5 hour (b) 6 hours (c) 7 hours (d) 8 hours of incubation at 37°C, respectively.
